# 1D “Spikelet” Projections from Heteronuclear 2D NMR Data—Permitting 1D Chemometrics While Preserving 2D Dispersion

**DOI:** 10.3390/metabo9010016

**Published:** 2019-01-16

**Authors:** Maryam Tabatabaei Anaraki, Wolfgang Bermel, Rudraksha Dutta Majumdar, Ronald Soong, Myrna Simpson, Martine Monnette, André J. Simpson

**Affiliations:** 1 Environmental NMR Center, Department of Physical and Environmental Sciences, University of Toronto Scarborough, Military Trial, Toronto, ON 1265, Canada; m.tabatabaeianaraki@mail.utoronto.ca (M.T.A.); ronald.soong@utoronto.ca (R.S.); myrna.simpson@utoronto.ca (M.S.); 2 Bruker BioSpin GmbH, Silberstreifen 4, 76287 Rheinstetten, Germany; Wolfgang.Bermel@bruker.com; 3 Bruker Ltd., 2800 High Point Drive, Milton, ON L9T 6P4, Canada; r.duttamajumdar@utoronto.ca (R.D.M.); Martine.Monette@bruker.com (M.M.); 4 Department of Chemistry, University of Toronto, 80 St. George Street, Toronto, ON M1C 1A4, Canada

**Keywords:** NMR, Metabolomics, ^1^H-^13^C Heteronuclear Single Quantum Correlation (HSQC), 1D-Spikelet

## Abstract

Nuclear magnetic resonance (NMR) spectroscopy is a powerful tool for the non-targeted metabolomics of intact biofluids and even living organisms. However, spectral overlap can limit the information that can be obtained from 1D 1H NMR. For example, magnetic susceptibility broadening in living organisms prevents any metabolic information being extracted from solution-state 1D 1H NMR. Conversely, the additional spectral dispersion afforded by 2D 1H-13C NMR allows a wide range of metabolites to be assigned in-vivo in 13C enriched organisms, as well as a greater depth of information for biofluids in general. As such, 2D 1H-13C NMR is becoming more and more popular for routine metabolic screening of very complex samples. Despite this, there are only a very limited number of statistical software packages that can handle 2D NMR datasets for chemometric analysis. In comparison, a wide range of commercial and free tools are available for analysis of 1D NMR datasets. Overtime, it is likely more software solutions will evolve that can handle 2D NMR directly. In the meantime, this application note offers a simple alternative solution that converts 2D 1H-13C Heteronuclear Single Quantum Correlation (HSQC) data into a 1D “spikelet” format that preserves not only the 2D spectral information, but also the 2D dispersion. The approach allows 2D NMR data to be converted into a standard 1D Bruker format that can be read by software packages that can only handle 1D NMR data. This application note uses data from Daphnia magna (water fleas) in-vivo to demonstrate how to generate and interpret the converted 1D spikelet data from 2D datasets, including the code to perform the conversion on Bruker spectrometers.

## 1. Introduction

Heteronuclear 2D NMR provides superior spectral dispersion over one-dimensional (1D) datasets. The 1D ^1^H spectra have been reported to have a peak capacity of ~3000, while that of 2D ^1^H-^13^C approaches 2,000,000 [1]. This becomes very important for the metabolomics studies of very complex samples that show poor resolution in 1D NMR (due to a combination of magnetic susceptibility distortions and sample complexity). As such, the importance of 2D NMR as a routine tool to extract otherwise inaccessible NMR information is continuing to rise [2,3,4,5,6,7,8]. 

Recently, stable ^13^C isotopic labeling [9,10,11,12] of organisms or live cells [13,14,15], has emerged as powerful approaches to overcome poor resolution and resonance overlap. However, in most of these studies 1D NMR, such as ^13^C NMR, is commonly acquired, as chemometric software packages that directly handle 2D NMR data are still limited [16,17,18,19]. 

The ^1^H-^13^C HSQC data, one of the 2D NMR techniques, are relatively well resolved and can be used to identify a range of metabolites in ^13^C labeled samples or concentrated non-enriched samples. The question now becomes: is it possible to preserve all the information from a 2D ^1^H-^13^C HSQC spectrum while generating a standard 1D format compatible with chemometrics and statistical packages? In the present application note, we describe the generation of such a format—1D spikelet—and demonstrate its applicability using both principal component analysis (PCA) and quantile plots (a useful visualization tool with the ability to identify changes within 1D datasets as color gradients) as examples. 

## 2. Results and Discussion

**Figure 1:**
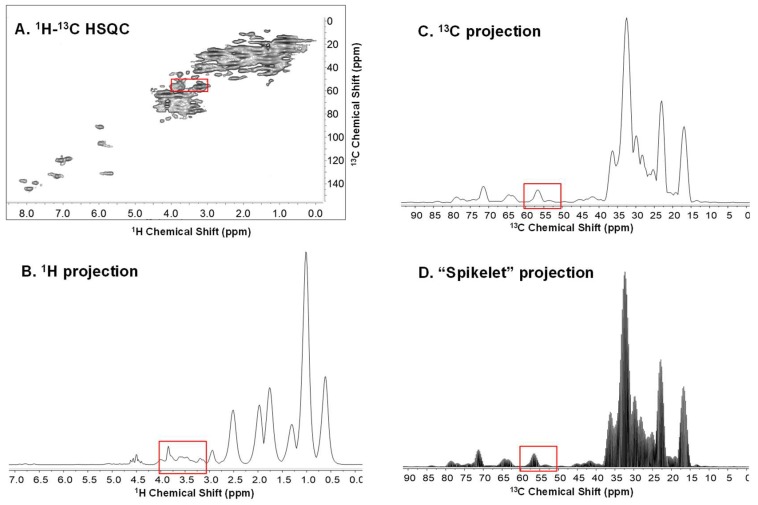
NMR data for ^13^C enriched in vivo *Daphnia magna* in a flow system [13]. (**A**) Full ^1^H-^13^C HSQC, (**B**) conventional ^1^H projection, (**C**) conventional ^13^C projection, and (**D**) spikelet projection.

**Figure 2:**
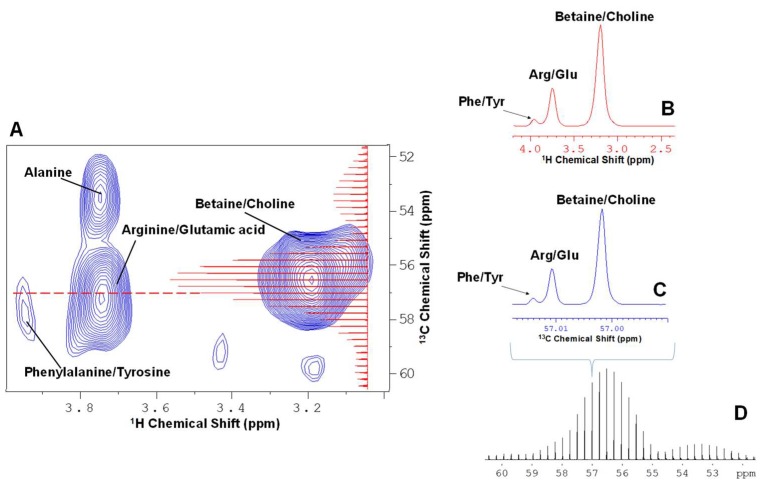
An expanded region around 57 ppm carbon, which demonstrates the information contained in the spikelet projection is identical to that in the full HSQC. (**A**) Expanded regions of 2D ^1^H-^13^C HSQC NMR, where three corresponding ^1^H signals from Betaine/Choline, Arginine/Glutamic acid, and Phenylalanine/Tyrosine are present; (**B**) indicates ^1^H chemical shift of the 57 ppm slice from the full HSQC, in which all three signals are well resolved; (**C**) shows ^13^C chemical shift of the exact same slice from (**D**) the spikelet projection.

The excellent spectral dispersion of HSQC helps metabolite discrimination, however the 2D format makes it incompatible with some chemometric approaches and software packages. Figure 1 compares the full ^1^H-^13^C HSQC (1A), conventional ^1^H projection (1B), and ^13^C projection (1C) against the information rich complete projection (1D) (termed here as “spikelet projection”) for in vivo *Daphnia magna*. Maryam Tabatabaei Anaraki et al. [20] have discussed the impacts of anoxic stress on *Daphnia magna*. In the study, it was demonstrated that the separation along PC1 arises from the accumulation of lactic acid, whereas the separation along PC2 arises from slight differences in the lipids between the 3 different populations of *Daphnia magna*. In the spikelet approach, complete ^1^H spectra extracted for each point in f1 (^13^C) in the processed spectrum are concatenated into a continuous profile, and then the corresponding ^13^C chemical shifts are projected onto the axis of the spikelet projection. The result is the overall profile of the spikelet projection and is analogous to the conventional ^13^C projection (Figure 1C), but for each ^13^C plane, the complete ^1^H spectrum is also preserved (see Figure 1D). Figure 2 demonstrates the concept of 1D spikelet projection with an expansion of the area around 57 ppm. Looking at the dashed line in Figure 2A (57 ppm carbon plane), three corresponding ^1^H signals from Betaine/Choline, Arginine/Glutamic acid, and Phenylalanine/Tyrosine are present. Figure 2B shows the 57 ppm slice from the full HSQC, in which all 3 signals are well resolved. Figure 2C shows the exact same slice from the spikelet projection (Figure 2D). The ^1^H information content is identical, with the only difference being the label on the axis (^13^C vs ^1^H). For cross checking assignments, the slice from the spikelet projection indicates where to look in the 2D (in this case 57 ppm carbon), after which proton information can be read from the original 2D data in the normal manner. It is important to reiterate that the advantage of the spikelet projections is that they are amenable to 1D data processing approaches. As such, methods that may only be available for 1D formats can be applied to information extracted from the 2D datasets that otherwise may not be possible. The complete code for converting 2D NMR data into the 1D spikelet format is available in Appendix A. The code is executed as a standard AU program in Bruker’s Topspin package. 

### 2.1. Principle Component Analysis (PCA) of the Spikelet Projections 

**Figure 3:**
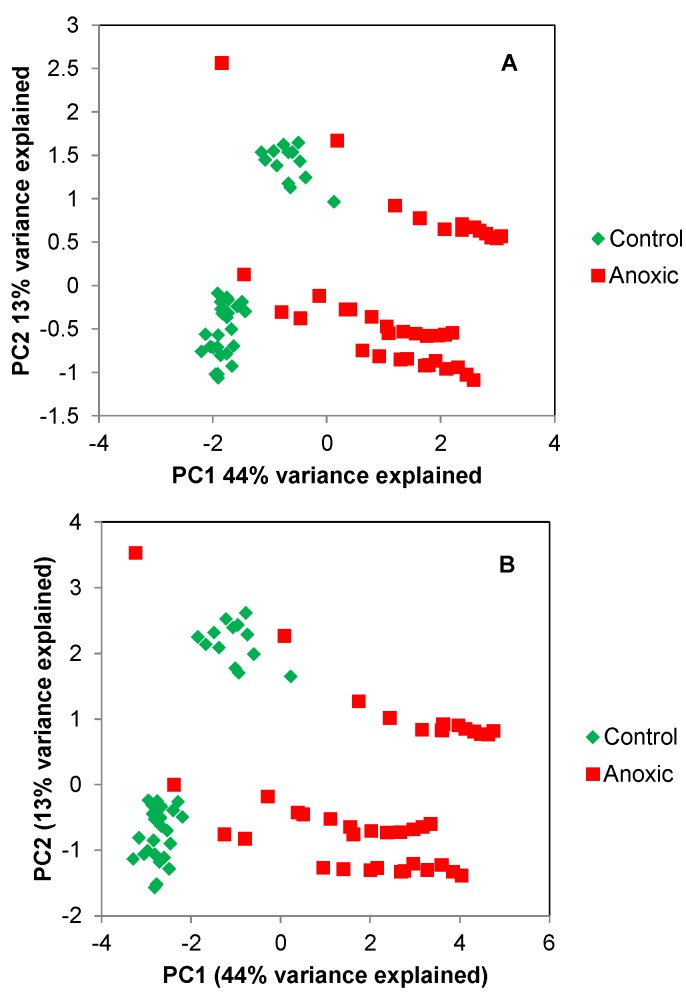
PCA plots (3 replicates) from the (**A**) full 2D dataset, and (**B**) the spikelet projections, offer near identical results.

Here, we simply demonstrate that the same result is obtained when a chemometrics approach is applied to the 1D spikelet data and 2D NMR data. Therefore, PCA analysis of NMR data of living *Daphnia magna* under decreasing levels of oxygenation is performed. Figure 3 compares PCA analysis using (A) the full 2D datasets, and (B) the spikelet projections, both performed in AMIX. The data from ^1^H-^13^C HSQC were obtained from 99% enriched ^13^C *Daphnia magna* (water fleas) with twenty organisms in the tube per replicate. HSQC spectra are collected every 1.5 h over a period of 24 h.

**Figure 4:**
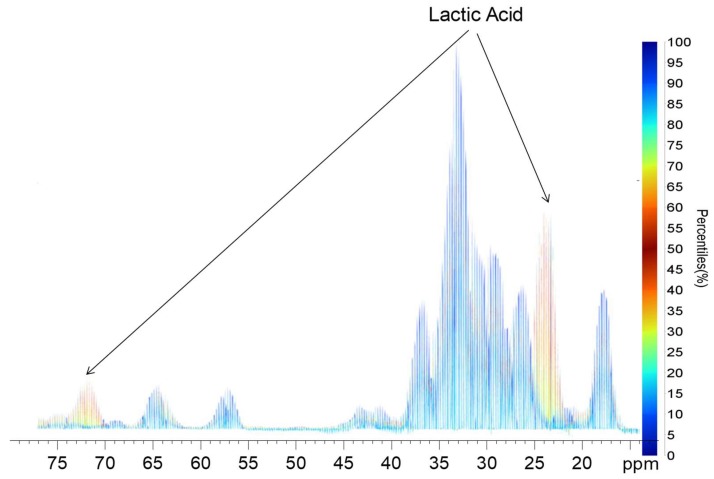
Quantile plot of 1D spikelet projection spectra generated from ^1^H-^13^C HSQC NMR. The red/orange peaks are from lactic acid that change over time, while other peaks in blue do not change.

For the control points (green), the organisms are supplied food and oxygenated water throughout the entire experiment, and the organisms are maintained in aerobic conditions (analogous to a conventional “control” dataset). The red dots represent a condition when the flow is stopped, and the organisms undergo anoxic stress that increases over time. Over time, as the anoxic stress increases (the red dots representing the stressed condition), distinct deviation from the control cluster (green dots) appears, due to variation caused by the build-up of lactic acid in the organisms’ metabolome (see quantile plot in Figure 4 for a simple way to visualize the lactic acid). With both the 2D data and 1D spikelet projections, the PCA shows near identical profiles (Figure 3A,B). There are some very slight variations between the PCA plots, likely due to the way Amix internally handles noise in the 1D and 2D during processing. However, the same information is obtained from each PCA plot. This is expected, considering the data being fed into the program are indeed identical and just displayed in a different format. 

### 2.2. Quantile Plot from the Spikelet Projections

Another interesting approach to visualize the change or variation across a time series is to use a quantile plot. To our knowledge, producing a quantile plot from the 2D NMR data is not possible with currently available software. Even if it were possible, the envelope describing the variation would be superimposed across a contour profile and would require visualization in 3D from a large number of angles to appreciate. However, by reducing the 2D data to 1D, a simple quantile plot is easy to construct (see Figure 4). Here, blue corresponds to signals that do not vary significantly across the dataset, whereas signals in the yellow, orange, and red vary the most across the data. It is very simple to assign the two main peaks of lactic acid, which are known to be the primary stress response of *Daphnia* under anoxic conditions. As such, quantile plots hold the potential to quickly evaluate what is changing across a series of data. While not normally applicable to 2D NMR, the 1D spikelet permits the implementation in an easy fashion. This is just a very simple example of how converting a 2D NMR data set into a 1D format may also open up new potential for analyses not directly applicable to the 2D data itself. 

## 3. Materials and Methods

### 3.1. Daphnia and Algae Culturing

Unlabelled *Daphnia magna* were cultured as previously described [21]. The neonates were isolated and exclusively fed a diet of ^13^C-enriched algae *Chlamydomonas reinhardtii* (Silantes, Munich, Germany) three times a week, during which 50–60% of the water was replaced everyday. The algae *Chlamydomonas reinhardtii* (not isotopically enriched), cultured as previously described [21], was added to the water reservoir to feed *D. magna* during the NMR experiments, in order to feed the organisms inside the NMR magnet without any additional signals from the food [6,7].

### 3.2. NMR Spectroscopy

All NMR experiments were performed using a Bruker Avance III HD 500 MHz (^1^H) NMR spectrometer with a 5-mm three channel ^1^H-^13^C-^15^N TCI Prodigy™ cryoprobe fitted with an actively shielded z-gradient. An external D_2_O lock bulb (~5 µL) was used for all experiments to keep the lock solvent separate from the organisms. The HSQC experiment was performed via double INEPT transfer using sensitivity improvement, States-TPPI phase cycling, and Garp4 decoupling. The SPR-W5-WATERGATE sequence was incorporated into the pulse-sequence to suppress the water signal [21]. A total of 128 increments were collected, each with 36 scans, 1024 time domain points, and recycle delay of 1 s. The interpulse delay during the INEPT transfer was based on ^1^*J*_HC_ of 145 Hz. Data were processed with a qsine function shifted by 90 ° in both dimensions. Data were zero filled to 2048 points in F2, while F1 was filled to 1024 points along with 32 coefficients of forward linear prediction. Spectra were calibrated against a range of known compounds in the Bruker Biofluid Reference Compound Database (v.2.0.0—2.0.5) prior to matching.

### 3.3. In Vivo NMR Spectroscopy

Fully ^13^C labelled *D. magna* were cultured by feeding them a ^13^C labelled diet from birth. Twenty of the 10-day-old *Daphnia* were placed in the 5 –mm NMR tube for each experiment, and 2D datasets were collected every 90 min over a period of 20 h. Three “control” datasets were obtained under continuous flow and the NMR tube was connected to a flow system, as previously described [13]. The temperature of the flow system and NMR was maintained at 10 °C for the duration of the experiment. The HPLC pump allowed for circulation of fluid containing food and water. Three “anoxic” experiments were conducted under no flow condition, and the organisms experienced anoxic stress that increased over time. An excess of ^12^C food was added (*Chlamydomonas reinhardtii*) to the NMR tube at the beginning of each anoxic experiment to ensure *Daphnia* will have enough food during the no flow conditions.

### 3.4. Data and Statistical Analysis

Principal component analysis (PCA) was performed on 2D and 1D spikelet NMR spectra using an Analysis of Mixtures (AMIX) statistics package (version 3.9.14, Bruker BioSpin). The 2D NMR spectra were binned using rectangular bins of 0.24 ppm in the ^13^C dimension (5 to 150 ppm) and 0.1 ppm in the ^1^H dimension (−2 to 12.5 ppm). The ^13^C buckets were calculated such that there was one bucket per processed point in F1. This gave rise to ~87,600 bins per 2D spectrum. The 1D concatenated spikelet data were binned with 0.1 ppm to give the same number of buckets. The approach using 2D NMR for PCA analysis has been introduced elsewhere [8]. The region 4.70–4.85 ppm along the F2 (^1^H axis) is excluded due to the residual H_2_O/HOD signals present. The “sum of intensities” was used as the integration mode and the scaling was set to “total intensity”. The quantile plot was calculated using AssureNMR (version 2.1, Bruker Biospin, Rheinstetten, Germany), using the 1D spikelet bucket table described above. 

## 4. Conclusions

This application note describes a simple approach to convert 2D ^1^H-^13^C HSQC NMR data into a 1D format (spikelet projection) that preserves the full information content and spectral dispersion. In turn, the approach potentially permits the larger array for tools that are available, or more developed for 1D NMR, to be applied, or where the visualization of higher dimensional data is challenging. The approach may have application beyond metabolomics. For example, processing of DOSY-HSQC data would require software to display the data in a 3D cube [22]. Converting the 2D HSQC data to a 1D spikelet format would still permit a conventional plot of chemical shift vs. diffusion to be performed. Similar arguments apply for the fitting HSQC collected with a series of T1 and T2 weighting for relaxation analysis, which is often performed in biomolecular NMR [23]. Conversion of the 2D HSQC format to a 1D format should permit additional analysis of the data when software capabilities required to work directly with the 2D format are not available. Furthermore, the conversion of 2D to 1D format may facilitate easier storage in universal formats that have recently been recommended for databased NMR-based metabolomics data [24]. This is particularly useful for the metabolomics in very complex mixtures or in-vivo data, where the additional dispersion of 2D NMR is required to reduce signal overlap. 

## Appendix A

Below is the code for an AU program to run in Bruker topspin. Paste everything below this line into a text file and store in the topspin installation path \exp\stan\nmr\au\src\user. Run from the HSQC data set that needs to be converted into a “Spikelet Projection”

AUERR = create_1d_from_2d(curdat, cmd);

QUIT

#undef MASR

#undef SINO

#undef TILT

#include <pstruc.h>

#include <pstruc_all.h>

#include <lib/par.h>

static struct all_pars p_f2s =

#include <pinit_all.h>

int create_1d_from_2d(const char* curdat, const char* cmd)

{

char infile[PATH_MAX], outfile[PATH_MAX], path[PATH_MAX];

char nuc1[10];

double sw_p1, sf1;

float axis_offs1, sr1;

int i,j,k,m;

int byteorder, ret;

int si1, si2, xdim1, xdim2;

int curprocno, nprocno;

int *rowin, *rowout;

int *dbuf;

FILE *fpin, *fpout;

/***** get dataset and parameters *****/

GETCURDATA

FETCHPARS("SI",&si2);

FETCHPAR1S("SI",&si1);

FETCHPARS("BYTORDP",&byteorder);

FETCHPARS("XDIM",&xdim2);

FETCHPAR1S("XDIM",&xdim1);

curprocno = procno;

nprocno = procno + 10000;

/***** read axis parameters *****/

FETCHPAR1S("SF",&sf1);

FETCHPAR1S("SW_p",&sw_p1);

FETCHPAR1S("OFFSET",&axis_offs1);

FETCHPAR1S("SR",&sr1);

FETCHPAR1S("AXNUC",&nuc1);

/***** create 1D *****/

WRPPARAM(nprocno)

DATASET(name, expno, nprocno, disk, user)

STOREPAR("PPARMOD", 0);

STOREPARS("PPARMOD", 0);

/***** allocate memory *****/

if ( 2. * (double)si1 * (double)si2 * (double)sizeof(int)

                    >= 2. * 1024 * 1024* 1024 )

 {

 (void)sprintf(path,"amount of memory requested too large\n");

 STOPMSG(path);

 }

dbuf = malloc ( 2 * si1 * si2 * sizeof(int) );

if (dbuf == 0)

 {

 (void)sprintf(path,"cannot get enough mermory\n");

 STOPMSG(path);

 }

rowin = dbuf;

rowout = dbuf + si1*si2;

/***** read 2D *****/

DATASET(name, expno, curprocno, disk, user)

(void)strcpy(infile, PROCPATH("2rr"));

fpin = fopen(infile,"rb");

if ( fpin == NULL )

 {

 (void)sprintf(path,"cannot open 2rr file: %s",infile);

 STOPMSG(path);

 }

(void)fclose(fpin);

fpin = fopen(infile,"rb");

fread(rowin,sizeof(int),si1*si2,fpin);

local_swap4(rowin,sizeof(int)*si1*si2,byteorder);

(void)fclose(fpin);

/***** sort(no xdim) *****/

for (m=0; m < si1/xdim1; m++)

 {

 for (k=0; k < si2/xdim2; k++)

  {

  for (j=0; j < xdim1; j++)

   {

   for (i=0; i < xdim2; i++)

    {

    rowout[i+j*si2+k*xdim2+m*si2*xdim1] = rowin[i+j*xdim2+k*xdim2*xdim1+m*xdim1*si2];

    }

   }

  }

 }

/***** store 2D *****/

strcpy(outfile, NEWPROCPATH(nprocno, "1r"));

fpout = fopen(outfile,"wb");

local_swap4(rowout,sizeof(int)*si1*si2,byteorder);

fwrite(rowout,sizeof(int),si1*si2,fpout);

(void)fclose(fpout);

/***** store parameters *****/

DATASET(name, expno, nprocno, disk, user)

STOREPAR("SI", si1*si2)

STOREPARS("SI", si1*si2)

/***** store axis parameters *****/

STOREPARS("AXNUC", nuc1);

STOREPARS("SR", sr1);

(void)strcpy(path, PROCPATH("procs"));

if ((ret = getpar(path, "$PROC", &p_f2s)) < 0)

 {

 Proc_err(DEF_ERR_OPT, "getpar failed on %s\n%s", path, par_err(ret));

 return -1;

 }

p_f2s.SF = sf1;

p_f2s.SW_p = sw_p1;

p_f2s.OFFSET = axis_offs1;

if ((ret = putpar(path, "$PROC", &p_f2s)) < 0)

 {

 Proc_err(DEF_ERR_OPT, "getpar failed on %s\n%s", path, par_err(ret));

 return -1;

 }

/***** view data *****/

VIEWDATA

Proc_err(ERROPT_AK_NO, "transpose finished");

return 0;

}
